# On the Lymphatic System

**Published:** 1883-10

**Authors:** W. Godfrey Dyas


CHICAGO MEDICAL

JOURNAL & EXAMINER.

VOL. XLVII.—OCTOBER, 1883.—NO. 4.

### Original Communications.

### ARTICLE I.

ON THE LYMPHATIC SYSTEM. By W. GODFREY DYAS, F.R.C.S. (Read before the Chicago Medical Society.)

Some years have now elapsed since I had the honor of reading before the Physical Society of Dublin, then existing, a paper on the subject which this evening will occupy our attention. On that occasion none could foresee all the light that has since been shed on a subject that still requires further illumination. To the microscope and chemistry are we indebted for many advantages not enjoyed by those who preceded us, though they brought to the task of investigation minds of a superior order, improved by discipline and culture. Yet with all the aid we have derived from advanced science and art, much remains for future investigation. Before I proceed to the consideration of the diseases of the lymphatic system, it may not be uninteresting to pass rapidly in review the opinions of former inquirers, as well as these of more recent times, regarding the anatomy of this system. And as we take a retrospect of the past from the fancied eminence we now occupy, we must not suppose the survey presents nothing but a barren and unprofitable scene; although we must acknowledge that our ignorance still of some of the more important points of lymph, its vessels and glands in a state of health, serves as an obstacle to any very extensive pathological inferences. Under the Ptolemies, in Egypt, the first discovery of the chyliferous vessels was made by Erasistratus. In the vivisection of kids he observed them, and mistook them for arteries. Hierophilus, an anatomist also of the Alexandrian school, saw them. He traced them from the intestine to their termination in the mesenteric glands, and concluded they belonged to the venous system. The existence of this portion of the lymphatic system was tacitly acknowledged by men of science, although uninformed with regard to its office until the time of Galen, who opposed the hitherto received opinions of the Alexandrian school. He omitted the necessary condition of vivisection, not being aware that chyle flowing along its vessels so as to present its milky hue, must be seen, if seen at all, during digestion, and at the moment of opening the abdomen of a living animal. From physiological considerations, based on the fact that the general system was supported by the ingesta of the digestive apparatus, and by the additional fact of the diameter of the tributaries of the vena porta far exceeding that of the corresponding arteries, Galen constructed a theory, which, unlike most of our thories, prevailed for fourteen centuries. He supposed that the nutritive fluids were drawn from the surface of the intestines by the radicles of the mesenteric veins, which carried them to the liver, whose special office it was to convert them into blood. Thus until the sixteenth century this theory held undivided sway, and by the authority of the promulgator, in addition to its plausible simplicity, paralyzed the efforts of future investigators.

At length, in 1532, Massa and Gabriel Fallopius, of Pisa, discovered lymphatic vessels—the former those of the kidneys, though he could not trace them to their origin or termination; the latter those of the liver, which he followed to their termination in their glands. In a few years subsequently Eustachius discovered the thoracic duct in the horse, and though having followed it in its course along the spine and through the diaphragm, he acknowledged that *there* he lost sight of it, and could not say how it terminated. None of thess inquirers knew what were the functions of these vessels; and matters thus remained until Aselli again saw the chyliferous vessels, remarked the similarity of their contents to those on the surface of the intestine during digestion, and inferred that these vessels were the absorbents of the chyle. He was aware that they traversed the glands, but supposed they ultimately terminated in the liver. Though he denied absorbent power to the radicles of the portal system, he believed in the elaborative functions of the liver in the conversion of the chyle into blood. Hitherto anatomists relied on the dissection of the lower species of the mammalia for what they advanced respecting the lymphatic system, but in the early part of the seventeenth century Gassendi, for the first time, saw in an executed criminal the lacteal vessels.

The next link in the chain of discovery was added in 1649 by Pecquet, who traced the connection between these vessels and the thoracic duct, and saw that the contents of this duct passed uninfluenced by the liver in their course to the subclavian vein. Subsequent investigation showed that the lacteal vessels were but a part of an extensive system of vessels, spread over and through the body, and all having the same absorbent function. In 1649 the lymphatics of the liver were discovered, together with their connection with the thoracic duct; and in 1651 Rudbeck discovered lymphatics in the pelvis, thorax, and in the lungs. In short, so fortunate was he in his researches that he felt justified in establishing his right as the discoverer of an independent vascular system. It was in 1787 that Mascagni published his great work, entitled, "Vasorum Lymphaticorum Corporis Humani Historia et Iconographia," with plates of this system. During the previous year Cruikshank published his work in London, "The Anatomy of the Absorbing Vessels of the Human Body." About the same time Jno. Hunter directed his powerful intellect to the elucidation of its functions, concluding that it *exclusively* presided over absorption. This for many years was the prevailing doctrine, till Magendie in France, and Tiedeman and Gruelin in

Germany, proved by the most convincing experiments that the lymphatics were *not* the sole instruments of absorption, but that the venous system participated in this function. In 1833 Panizza, for the first time ("Osservazioni Sulle Vasi Linfatitici, Pavia;" and at the same time appeared Formann's work, "Mémoire Sur les Vaisseaux Lymphatiques, Liège"), demonstrated the networks from which arise the lymphatic vessels—a wonderful triumph of art! For from the anatomical arrangement of valves in the lymphatics of man, which are comparatively strong and remarkably numerous, it was found impossible to reach these networks by injection indirectly from the trunks, and it therefore required the most delicate instruments in the hands of experienced manipulators to directly inject the network itself.

This has been done perhaps more successfully by Sappey than by any other anatomist. He has most satisfactorily shown how unequally distributed this system is—has incontrovertibly demonstrated how many anatomists have been deceived, supposing they had injected a network. when they had forced the fluid of injection, or mercury, as it might be, into the cellular tissue, and imagined they had succeeded in filling the lymphatic capillaries of the tissue. He remarks that this occurs by not having the instrument for injection sufficiently fine in the point, and also by passing it in too deeply. Owing to these two errors, the capillaries of lymphatics, of veins and arteries, are opened and the mercury may pass, he says, into each of these orders of vessels, and even penetrate the cellular tissue.—"It is extremely rare, however, for the metal to pass into the arteries, but it is frequently seen entering the veins." Now, it must be remembered how a subcutaneous lymphatic network lies relatively to the capillary system of blood-vessels—always superficial to it. This fact in a pathological sense is a matter of importance to know, for I hope to show that a network may be affected by diffuse inflammation without the underlying capillary system of blood-vessels being to the same extent necessarily involved in the affection.

This arrangement of the lymphatic vessels being superficial to the capillary system of the blood-vessels, is the rule as regards the cutis and such mucous membranes as have a pavimentous epithelium. Where the epithelium is cylindrical, thc sanguineous network is superficial. This latter arrangement is well marked in the villi of the small intestines, where the chyliferous vessel in the center of the villus is surrounded by a network of minute capillaries of the sanguineous system. Whereas, the mouth, pharynx and œsophagus present a pavimentous epithelium and a lymphatic capillary network superficial to the sanguineous network. Again, the stomach is lined by a cylindrical epithelium and the vascular network of blood-vessels is superficial to the lymphatics. So far, I am unable to find an exception to this arrangement as a general rule, unless that which obtains in the lungs, where in the bronchioles and cells the vascular network is separated from the atmosphere by an extremely thin layer of pavimentous epithelium. (Bert.)

Should we attempt to carry our inquiries to the source of these networks, we trace them to radicles most difficult to see, even by the aid of the microscope and nitrate of silver injections. In the villi of the small intestines they are more readily seen than elsewhere, and as there they end each in a cul-de-sac, we infer the nutritive juices enter them by endosmosis. It is now a well ascertained fact that they do not commence by open orifices on the surface of the mucous membrane of the intestine. Brucke, of Vienna, in his work, "Ueber die Chylusgefässe, und die Resorption des Chylus," insists upon there being no proper wall at the base of an epithelial cell of a villus of the small intestine—that there is merely a kind of border circumscribing the orifice, which is habitually closed by a mucous plug, and that through this plug fatty matters enter the chyliferous vessel. The difficulty now would be, how to get out of the epithelial cell; but Brucke maintains the apex of the cell is pierced with an opening which permits the fat to reach the sarcodic tissue of the villus, whence it easily makes its way to the chyliferous vessel; this vessel, according to him, having no proper wall, cannot therefore present an obstacle to its entrance. Wittich, Milne-Edwards and Lambl are disposed to take the same view—which, I need scarcely add, is not entertained by our profession generally. That a selective property exists in the mucous membrane is inferred from the fact that if the poison of serpents or woorara, even in the slightest quantity, be introduced into the system by wound, the result may be fatal, yet either may be introduced within the alimentary canal and be innocuous. The following experiment made with woorara in 1850, as published in *L'Union Médicale*, p. 125, is to the point:

The gastric mucous membrane of a recently killed animal was adapted to an endosmometer, so that the mucous membrane looked outward and the endosmometer containing sugared water was then placed in a watery solution of woorara; endosmose took place in between three and four hours, for the liquid rose in the tube and yet contained no trace of woorara, as was ascertained by endeavoring to inoculate with it. If the experiment were allowed to go on for a much longer time, the endosmosis of the poison might occur; but we should then find that the mucous membrane had undergone modification, the mucus and epithelium covering it being altered so that imbibition and endosmosis of the woorara becomes possible. And if, in place of taking a quite fresh mucous membrane we take one that has undergone some change, the endosmosis of the poisonous fluid occurs instantly. It moreover appeared that all mucous membranes of the body, excepting that of the lungs, enjoy this immunity. When the poison was applied to this, the pulmonary mucous membrane, the effects were the same as if the application were made to subcutaneous cellular tissue. This is Kölliker's opinion as given in his work—Mikroskopische Anatomie, Band 2d, p. 169. This is the opinion generally held by the best observers, among whom we must rank Dr. Carpenter, whose words I have just now quoted. Now, the readiness with which the absorbents of this last mentioned tissue always receives the poison, as compared with the resistance of all mucous membranes to its reception, excepting that lining the bronchi, proves that the selective power is in the mucous membrane, not in the absorbents; and, moreover, the fact that this resisting power resides in the membrane of the animal recently killed, but is not retained after three or four hours, satisfactorily proves that this selective power is not merely a physical phenomenon, but is a vital process. When we examine the internal surface of the intestinal mucous membrane of a recently killed animal, in which the absorption of nutritive matter is in full action, we observe the lacteals turgid with chyle, their extremities imbedded in numerous globules, presenting an opalescent appearance and giving to the end of the villus a mulberry-like form. This appearance is due to the epithelial cells covering the extremity of the villus, with the chyle which they have absorbed and which they afterward yield up to the lacteals. In this connection it is right that I should state that Professor Delafield maintains that connective tissue contains a very abundant system of lymphatics, consisting of canals of different sizes and of stellate spaces communicating with them. Dybokowsky and Klein describe lymphatics of the pleura. Delafield represents them in these plates: iv, vii and viii. In the fourth plate we see the pleura magnified 750 diameters; in plate vii, lymphatics of the parietal pleura of the dog magnified 90 diameters; in plate viii, lymphatic spaces and connective-tissue cells magnified 750 diameters. It is not now my intention to dwell on the discrepant opinions of anatomists regarding the existence or non-existence of the lymphatic system in the several organs; let it suffice to say that lymphatic vessels have been denied to the brain and spinal marrow, to the free surfaces of serous membranes, to those of synovial membranes, to the cornea, to the connective tissue; and, though it is conceded that the mucous membranes are generally supplied with lymphatic vessels, it is on high authority stated that the lining membrane of the uterus, of the bladder and of the conjunctiva, both palpebral and ocular is without them. Even in the cutaneous system, which in some places is richly supplied, there is nothing like a uniform distribution of them. The parts most distant from the center of the circulation especially abound in them; the fingers and toes, the scrotum, the glans and prepuce, the extremity of the nose, the pavilion of the ear, and the scalp, are the points where they can be seen in abundance. To these may be added the secreting glands, especially the mammary gland, the testes, the ovaries, the muscular layer of the gravid uterus, the liver, the lungs. The internal or lining membrane of the heart possesses lymphatics, but that lining arteries and veins does not present any appearance of them. Those among us who have seen Mascagni's plates, in which he represents the subconjunctival membrane as a rich network of lymphatics, prepossessed as he was in favor of his theory, that the cellular tissue was the matrix of the lymphatic system, cannot avoid being struck by the apparent adaptation of the representation to the theory. Yet if we are to believe anatomists of the present day who have made repeated experiments to test this theory, we are assured that the views of Mascagni were illusive and unsupported by a single fact. Notwithstanding this, recently the German School, including such names as those of Virchow, Teichmann Ludwig, Tomsa, His, Frey and Recklinghausen, have adopted Mascagni's views as to the connective tissue being the source of the lymphatic system, though differing, respecting the mode of its origin in relation to the tissue. These men have been challenged to demonstrate this origin, but to the present they have been unable to do so. The same proof of Robin's lymphatic sheath, surrounding as with a muff the capillaries of the brain, has been demanded, and with the same success. Neither he nor His, who calls them the perivascular canals, has been able to trace them to an origin or to a termination in a lymphatic gland—a necessary condition of every lymphatic vessel—as none such in man can reach the general circulation without passing through at least one gland. Without going more into detail, we may group the prevailing opinions with regard to the origin of the lymphatics under three heads: first, The lymphatics are closed at their origin; second, They are not closed, but communicate with the corpuscles of the connective tissue ; and third, They are not closed, but communicate with what Recklinhausen calls the plasmatic channels. Now, Recklinhausen does not deny the existence of these corpuscles of the connective tissue, but places them in relation to the origin of the lymphatics in his lacunæ channel, or slits of the fibrillary substance of the connective tissue. Perhaps we may receive as a dogma the position now about to be laid down, as it rests on the authority of the most distinguished anatomists of the present time.

The lymphatic vessels take their origin in a system of most minute capillaries and lacunæ, which unite to form capillaries of greater magnitude, then trunks. These lacunæ are stellate spaces, the points of which anastomose with those of other lacunæ.

The minute capillaries referred to above are extremely fine canals which unite the lacuna to each other. These minute capillaries form loops in anastomosing with the sanguineous system of capillaries, thus communicating with the general circulation of the sanguineous system. The diameter of these minute capillaries is such that nothing can enter them from the blood-vessels but serum. The lymphatic system not only concurs to absorption—it has the office of forming the solid parts of the blood. The lacunæ above mentioned are filled with very fine granulations, the first rudiments of future white or colorless corpuscles, and these are carried along by the larger lymphatic capillaries. It may now be asked, are not the several bodies which under various names are termed the hæmopoietic organs, the special organs by which these colorless corpuscles are formed? That they are not the only source of these corpuscles is evident from the fact that these are seen at the distal end of the lymphatics before these vessels meet with a gland. That they exist independently of lymphatic ganglions appears from the existence of them in the blood of some of the vertebrata that possess a lymphatic system but no ganglions. There are many sources of these corpuscles, and disease may affect an organ whose office it is to produce them without necessarily involving the supply from other sources. If we have had much divergence of opinion in regard to the origin of lymphatic vessels, we have had quite as much respecting the anatomy and functions of the lymphatic glands. Upon some points anatomists generally are agreed. In the first place it is allowed that each lymphatic gland is enveloped in a fibro-cellular capsule, that sends processes from its internal surface, dividing it into areola of varying size and shape, as these processes converge towards the center of the gland—that these areolæ contain follicles, which, with their contents of corpuscles intermixed with a delicate network of connective tissue, constitute the parenchyma or proper substance of the gland; that the afferent and efferent lymphatic vessels maintain the most intimate relations with this parenchyma, and that finally each gland is supplied in its interior with blood-vessels and nerves. In matters of detail there is, however, considerable difference of opinion. It is contended by some (Le Denteu and Lorguet) that the afferent vessels immediately on passing through the fibrous capsule divide themselves into a network of capillaries formed of merely their epithelial layer lined by a thin wall of laminated tissue. From this network pass special ducts called sinuses, which penetrate deeply in maintaining relations with the blood-vessels on the one hand, and with the fundamental tissue, whether termed the proper parenchyma or adenoid tissue, on the other. The blood-vessels are closely united to the tracts of laminated tissue derived from the capsule. Vessels and tracts are completely surrounded by a cylinder, of which they occupy the center, and this cylinder is the sinus. Its internal surface is united to the external surface of the vessel and of the fibrous tract by a delicate network of connective tissue.

From its external surface a similar network passes to the parenchyma, in the midst of which it is plunged. The blood-vessels, as well as the tracts of laminated tissue by which the former are supported, occupy the axis of the sinus. The perpendicular cut of this has the form of a ring. The lymph circulates in the sinus between the central axis and the wall of the sinus. All the surfaces which are in contact with these parts; viz., the external surface of the vessel and that of the central axis—the internal surface of the wall of the sinus—the network of connective tissue uniting the latter to the axis, are covered with an epithelium of elongated cells. The same epithelium covers the connective tissue of the parenchyma. At about the third of the thickness of the gland, the sinuses terminate in another network; from this the efferent vessels arise. These no longer maintain the same relation with the blood-vessels and fibrous tracts.

These are the views that principally prevail at present on this subject. Robin, however, denies that the sinuses communicate with the proper substance of the gland. Others maintain that this communication is easily proved by means of injecting the corpuscles in the afferent lymphatics—that these corpuscles are found not only in the sinuses and in the epithelial cells that cover them, but also in the epithelial cells of the adenoid substance. To this it is objected that the injection proves nothing—that the presence of the colored corpuscles in the epithelium of the sinus is the result of mechanical penetration, and that the infiltration of the epithelium of the trabeculæ of the proper substance is consecutive to the passage of the corpuscles through the wall of the sinus, and that it is not owing to the circulation of lymph in the proper substance itself.

The view of the distinguished Professor of Anatomy to the Faculty of Medicine of Paris does not coincide with the above. He says that in the first place the intimate texture of the glands is identical at all points of their thickness; that they comprise a fibro-cellular frame-work, constituted by an enveloping capsule and numerous elongations which extend from the latter toward the central part; secondly, a parenchyma or proper substance; thirdly, lymphatic vessels afferent and efferent, which maintain the closest relations with the substance; and, finally, blood-vessels and nervous filaments. The prolongations of the fibro-cellular capsule are very different in form and dimension. Some represent partitions, others are thicker, or round and filamentous. All these prolongations extend from the circumference of the gland to its center in continually intercrossing, circumscribing areolæ of unequal and irregular dimensions, and becoming smaller as they approach the center. The proper substance consists of closed vesicles like those observed in the thymus, thyroid body, and the spleen. These vesicles vary in size, being susceptible of considerable increase under the influence of hypertrophy, to which these glands are very liable.

The membrane which forms them is very thin, homogeneous, soft and friable. It is lined, or rather filled, with a spherical nuclear epithelium, with which are frequently mixed pavimentous epithelial cells of variable size, containing a large nucleus. When these vesicles are opened they allow their epithelium to escape in abundance. They occupy the areolæ that are circumscribed by the fibro-cellular prolongations, which areolæ are themselves traversed in every direction by filaments of the finest texture. These filaments present a reticulated arrangement, hence the term reticulum, by which they are frequently designated. They thus appear to be a part of the fibro-cellular network of the gland, of which the fibers isolate themselves in order to form a still more delicate frame-work containing in its meshes the element of the proper substance. This substance represents the active element of the glands. It is to it that is confided the office of reacting on the chyle and lymph, and of assimilating them, or at least of rendering them more and more assimilable to the blood, of which they are required to constitute a part. The afferent vessels divide before plunging into the gland; their first divisions creep over its surface; after a short course they pierce the fibrous capsule, then penetrate the superficial layers, where they subdivide and advance towards the deeper parts in proportion as they become smaller. These branches and their ramifications follow the prolongations of the fibro-cellular framework in advancing to the recticulum, and by their last divisions come in close relation with the proper substance. It is from the terminal extremities of these divisions that arise the first radicles of the efferents. In thus being continuous, the afferent and efferent vessels form a plexus before the latter leave the gland. This continuity may be seen in the embryo at a time when the glands are anatomically less complicated than in a more advanced stage of development. An argument in favor of this continuity may also be adduced from comparative anatomy; for, as we ascend in the scale of animated nature, we meet with no lymphatics until we arrive at the division vertebrata, and then, for the first time, in fishes we see lymphatics without glands or valves; but, in lieu of glands, we see plexuses. In the same way in reptiles we see no glands, but for the first time we see valves in a rudimentary state. In birds, though as yet the glands are not as yet fully developed, glands may be observed in the cavity of the thorax and at the base of the neck; elsewhere their place is supplied by plexuses. At length in the mammalia, in which class this symptom reaches its full development, the plexuses give way to glands. It is evident from this that in fishes, without leaving its vessels, the lymph in the lower class of vertebrata is modified in its passage through them; that in them no intervening organs are necessary to its elaboration. In a more advanced class we have further evolution in plexuses, still there is continuity of vessels. There is no interposition of cells, and yet it is to be inferred there is no want of such additional apparatus for the fulfillment of the functions of this system. Finally, on injecting a gland and drying it you cannot see sinuses, but canals or portions of canals interlacing, like vessels in a plexus, with each other. Again, as we examine organized structures we cannot help remarking the simplicity and harmony that nature affects. Even where there is a variety of purpose you may often see a unity of design, and unless we can prove some special modification of function, we have no right, logically, to assume a deviation from the conformity to a general plan.

The lymphatic system, as all are aware, in man terminates in two trunks—one the thoracic duct, which, commencing at the second lumbar vertebra, passes to the left side of the neck, and forming there an arch, opens in the left subclavian vein, where it unites with the internal jugular; the other, the great right lymphatic vein, terminates in the R. subc. vein, where this unites with the internal jugular.

We may observe here a remarkable adaptation of means to ends, in the mode in which these two trunks discharge their contents into the blood-vessels—the convergence of the veins at an angle affording a more facile exit to them than they could have at any other point.

It may now be enquired if the lymphatic trunks communicate with the venous system directly elsewhere, and do their minute capillaries in the glands directly unite with the capillaries of the blood-vessels. We will first consider the former question.

I believe the opinion generally received is, that as we ascend in the scale of the vertebrata, the communication of the lacteal vessels with the veins becomes more and more restricted, until we arrive at the class mammalia, to which the following characteristics may be assigned: 1st. Greater development of valves. 2d. Increased number of glands. 3d. More limited communication with the venous system. Few anatomists of the present time will maintain the communication of a lymphatic trunk with the venous system elsewhere than in the junction of the subclavian and internal jugular veins; though Stene, Wepper, Schmiedd, Boerhaave and Meckel state they saw the junction with either the cava, vena azygos, hypogastric, lumbar veins or vena porta. On the other hand, we have a host of authorities denying the communication with other veins than the subclavian and internal jugular, such as Haller, Cruikshank, Mascagni, Fohmann, Panizza, Rossi, Blandin, Cruvelhier, Carpenter and Sappey. Those in favor of the former opinion rest their advocacy of it on the acknowledged free communication between the trunks of the two systems in the lower classes of the vertebrata. Thus in fishes this communication has been satisfactorily demonstrated by Fohmann. This is well seen in the eel, where the caudal sinus, the rudimentary representation of a more advanced type of organization empties itself into the caudal vein, the orifice of which is provided with a valve. Again, in reptiles, which possess a very extended lymphatic symptom, this is supplied with contractile cavities called lymphatic hearts, which collect the lymph and directly pump it into veins. This provision for sending forward the lymph is remarkably well observed in frogs, which have two pairs of these pulsating cavities. In birds, also, in addition to their two thoracic ducts terminating in the angle of the subclavian and jugular veins, their lymphatics have also direct communication with the veins of the lower extremity. We should not, however, be satisfied with any argument derived from the structure of inferior animals, in a question that is susceptible of demonstration, as this certainly should be, and when we know that in this matter sub judice, the anatomists denying the communication, have formed their opinion, aided by improved methods of dissection and preparing, we cannot hesitate to reject the views of the advocates of multifarious communication.

However, though there is no doubt that the usual termination of the thoracic duct is in the angle of the jugular and subclavian veins; yet a deviation occasionally exists in the human subject, now and again presenting a plexus rather than a single vessel, and terminating in the veins by several apertures, or even sending off a branch, as in the hog, to terminate in the vena azygos.

The next question to engage our attention will be, Do the lymphatic and venous systems communicate directly in the lymphatic glands? Tiedeman and Fohmann, both great authorities, think so, and rest their opinion on the following facts: The marked contrast between the number and size of the lacteal vessels and the small caliber of the thoracic duct. The continuation of life when this duct has been obliterated. The passage of chyle into the sanguineous system, when the principal mesenteric glands are affected by tubercular degeneration; provided that those nearest to the intestinal canal are unaffected by disease; and the absence of general or partial dropsy when the mesenteric glands are thus diseased. Again, it is stated that the venous capillaries can be injected in the glands from the different vessels. But this is denied by Cruveilhier, Carpenter and Sappey. When the injection thus pushed is seen in the veins, it is the result of a rupture which easily takes place when the gland is softened from commencing decomposition; but in every instance where the injection was made in glands fresh and free from decomposition, the efferent vessels have been filled, but not the venous capillaries.

I have now hastily glanced at some of the most important characteristics of the anatomy of the lymphatic system, not intending that any such cursory treatment of my subject should be considered as an effort at its exhaustive exposition, or more than a necessary introduction to the treatment of those morbid conditions of the lymphatic vessels and glands that remain for future consideration. Neither a wide expansion of general views nor a detailed precision of individual facts would be consistent with my aim, which has been to present to the profession those leading anatomical points that may conduce to a more accurate conception of views on the pathology and symptoms of diseases of the lymphatic system.

